# Effects of Heat-Killed *Bifidobacterium breve* B-3, a Postbiotic, on Muscle Mass-Related Parameters in Patients with Sarcopenia: A Randomized, Double-Blind, Placebo-Controlled Pilot Trial

**DOI:** 10.3390/nu18183074

**Published:** 2026-09-20

**Authors:** Daisuke Asaoka, Shin Yoshimoto, Noriko Katsumata, Noriyuki Iwabuchi, Naotake Yanagisawa, Toshitaka Odamaki, Jin-Zhong Xiao, Tsutomu Takeda, Shigeo Koido, Toshifumi Ohkusa, Akihito Nagahara, Nobuhiro Sato

**Affiliations:** 1 Department of Gastroenterology, Juntendo Tokyo Koto Geriatric Medical Center, Faculty of Medicine, Juntendo University, Tokyo 136-0075, Japan; 2 Biotics Research Institute, Morinaga Milk Industry Co., Ltd., Kanagawa 252-8583, Japann_katumt@morinagamilk.co.jp (N.K.); t-odamak@morinagamilk.co.jp (T.O.); 3 Medical Technology Innovation Center, Juntendo University, Tokyo 113-8421, Japan; n-yanagisawa@juntendo.ac.jp; 4 Morinaga Milk Industry Co., Ltd., Tokyo 105-7122, Japan; j_xiao@morinagamilk.co.jp; 5 Department of Gastroenterology, Faculty of Medicine, Juntendo University, Tokyo 113-8421, Japan; t-takeda@juntendo.ac.jp (T.T.); nagahara@juntendo.ac.jp (A.N.); nsato@juntendo.ac.jp (N.S.); 6 Division of Gastroenterology and Hepatology, Department of Internal Medicine, The Jikei University Kashiwa Hospital, Chiba 277-0004, Japan; shigeo.koido@gmail.com (S.K.); ohkusa@jikei.ac.jp (T.O.)

**Keywords:** sarcopenia, postbiotic, heat-killed *Bifidobacterium breve* B-3, MCC1274, skeletal muscle mass, bioelectrical impedance analysis, IGF-1, gut–muscle axis, randomized controlled trial

## Abstract

**Background/Objectives**: Sarcopenia contributes to frailty and functional decline in older adults; however, effective adjunctive strategies remain limited. Heat-killed *Bifidobacterium breve* B-3 (B-3HK), a postbiotic, has shown muscle-related effects in preclinical studies; however, clinical evidence in patients with established sarcopenia is scarce. This pilot trial aimed to evaluate these clinical effects. **Methods**: In this 24-week randomized, double-blind, placebo-controlled trial, adults aged 50–89 years with Asian Working Group for Sarcopenia (AWGS) 2019-defined sarcopenia received B-3HK (2 × 10^10^ cells/day) or placebo daily. The primary outcome was the change in knee extension strength at week 24. Secondary outcomes included body composition measured using bioelectrical impedance analysis (BIA), dual-energy X-ray absorptiometry (DXA), physical performance, Eating Assessment Tool-10 (EAT-10), serum biomarkers, gut microbiota, and safety. Secondary outcomes were exploratory with nominal *p*-values and no adjustment for multiple comparisons. **Results**: Fifty participants were randomized, and 49 were included in the full analysis set. Compared with placebo, B-3HK did not significantly improve knee extension strength. Exploratory analyses showed between-group differences favoring B-3HK in the BIA-derived skeletal muscle mass index, limb muscle mass, EAT-10 score, and serum insulin-like growth factor-1 and 25-hydroxyvitamin D levels. These differences were primarily driven by the attenuation of the decline in the placebo group and were not corroborated by DXA-derived indices or functional outcomes. The gut microbiota composition did not differ between groups. No serious adverse events were observed. **Conclusions**: B-3HK did not improve muscle strength in patients with sarcopenia. Exploratory analyses identified nominal between-group differences in body composition and endocrine outcomes; however, these findings were unadjusted for multiplicity and should be regarded as hypothesis-generating rather than confirmatory evidence of efficacy. These data may help inform the design of future adequately powered trials, but confirmation is required before any therapeutic benefit can be inferred.

## 1. Introduction

Age-related declines in the mass and strength of skeletal muscles are major contributors to sarcopenia, a condition that is strongly associated with frailty, disability, and increased mortality in older adults. Sarcopenia has emerged as a major public health concern in aging societies, and effective preventive and therapeutic strategies remain global priorities. Resistance exercises and nutritional optimization are central to sarcopenia management; however, their effectiveness may be limited in some older adults because of comorbidities, reduced adherence, or age-related anabolic resistance.

In recent years, increasing attention has been directed toward the gut–muscle axis as a potential modulator of muscle health. Gut microbiota-derived metabolites, such as short-chain fatty acids (SCFAs), influence skeletal muscle metabolism, mitochondrial function, and systemic inflammation through pathways involving AMP-activated protein kinase (AMPK) and mammalian target of rapamycin (mTOR) signaling. Observational studies have shown that alterations in the gut microbiota composition are associated with skeletal muscle mass index, muscle strength, and gait speed in older adults, supporting a potential link between intestinal ecology and sarcopenia [1].

Clinical trials and meta-analyses evaluating probiotic interventions have reported modest improvements in muscle strength and physical performance in older adults; however, their effects on muscle mass remain inconsistent [2]. Most previous studies have focused on community-dwelling older individuals or populations at risk of sarcopenia, rather than on patients with established sarcopenia. Consequently, clinical evidence regarding microbiota-targeted interventions in sarcopenic populations is limited.

*Bifidobacterium breve* B-3 (termed MCC1274) has attracted attention as a candidate strain that influences body composition. In a randomized controlled trial in pre-obese adults, compared with placebo administration, daily intake of *B. breve* B-3 for 12 weeks significantly reduced body fat and increased muscle mass, despite no changes in physical activity [3]. Recently, the interest in live probiotics has shifted to postbiotics. Postbiotics are non-viable microbial cells or their components that exert biological effects on the host, potentially through pattern-recognition receptor-mediated immune signaling, without requiring intestinal colonization by live organisms [4]. Compared with live probiotic formulations, postbiotics may offer practical advantages, including improved safety, stability, and reproducibility, to older adults.

Preclinical studies have demonstrated that heat-killed *Bifidobacterium breve* B-3 (hereafter referred to as B-3HK) activates anabolic and mitochondrial pathways related to skeletal muscle hypertrophy and energy metabolism, including Akt–mTOR and AMPK–peroxisome proliferator-activated receptor gamma coactivator 1-alpha (PGC-1α) signaling [5]. However, whether these mechanistic findings translate into clinically meaningful benefits for patients with sarcopenia remains unclear.

Moreover, clinical evidence of the effects of biotic interventions on muscle strength and mass in individuals with established sarcopenia is scarce. In particular, the effect of B-3HK on functional and body composition-related outcomes has not been adequately examined in randomized controlled trials.

Therefore, we conducted a 24-week randomized, placebo-controlled trial to evaluate the effect of B-3HK on knee extension strength in patients with sarcopenia while exploring changes in muscle mass-related parameters, physical performance, oral function, endocrine markers, gut microbiota composition, and safety outcomes.

## 2. Materials and Methods

### 2.1. Study Design and Ethics

This 24-week, randomized, double-blind, placebo-controlled, parallel-group trial was conducted in patients with sarcopenia. The study protocol was approved by the Juntendo University Certified Review Board (certification number: CRB3180012; protocol code: J20-013; date of approval: 10 November 2020) and registered in the Japan Registry of Clinical Trials (jRCTs031200200; date of registration: 13 November 2020). All participants provided written informed consent before enrollment.

### 2.2. Participants

The participants were outpatients who visited the Department of Gastroenterology at the Juntendo Tokyo Koto Geriatric Medical Center. The inclusion criteria were as follows: age 50–89 years; sarcopenia defined according to the Asian Working Group for Sarcopenia (AWGS) 2019 criteria, ability to walk independently, and provision of written informed consent for study participation. Sarcopenia was diagnosed using the diagnostic algorithm recommended by the AWGS 2019 consensus [6]. In brief, sarcopenia was defined as low appendicular skeletal muscle mass combined with low muscle strength and/or low physical performance. Handgrip strength was measured twice for each hand using a handgrip dynamometer (Toei Light Co., Ltd., Saitama, Japan), and the highest value was recorded as the maximum muscle strength. Low grip strength was defined as <28 kg for men and <18 kg for women, according to the AWGS criteria. Gait speed, manually assessed using a stopwatch, was defined as slow when <1.0 m/s according to the AWGS criteria. Lean and regional fat masses were assessed using whole-body dual-energy X-ray absorptiometry (DXA) (Prodigy Advance; GE HealthCare, Madison, WI, USA). Participants were positioned for whole-body scans following the manufacturer’s protocol. Whole-body fat mass and lean mass were divided into the arms, legs, and trunk. The appendicular lean mass was estimated as the sum of the lean masses of the upper and lower limbs. The appendicular skeletal muscle mass index (SMI) was calculated as appendicular lean mass divided by height squared. Low appendicular skeletal muscle mass was defined as an appendicular SMI < 7.0 kg/m^2^ for men and <5.4 kg/m^2^ for women.

Exclusion criteria included neurological disorders (including sequelae of head injury, Parkinson’s disease, Huntington’s disease, hydrocephalus, progressive supranuclear palsy, epilepsy, multiple sclerosis, and intracranial infectious disease), major depressive disorder, bipolar disorder, alcohol or other substance dependence, cerebral infarction, brain tumor, intracranial hemorrhagic lesions, dementia, neurosyphilis, exercise limitation due to joint or musculoskeletal disease, severe systemic disease, advanced gastrointestinal cancer, history of major gastrointestinal surgery, gastrointestinal disorders such as inflammatory bowel disease, regular use of medications or supplements affecting bowel motility, known or suspected drug or food allergies, excessive smoking or alcohol use, markedly irregular lifestyle habits, use of antidementia or psychotropic medications, severe diabetes mellitus requiring insulin therapy, habitual resistance training, and any other condition that could render study participation inappropriate, as judged by the principal investigator.

### 2.3. Sample Size Calculation

This was an exploratory study. As sufficient information was not available to estimate the target sample size based on statistical power, the sample size was determined using a precision-based approach. In a previous study [7], the between-group difference in knee flexion–extension strength (Nm/kg) at three months after nutritional intervention was estimated at 0.11 (95% confidence interval (CI): 0.11 ± 0.09; width: 0.17). Furthermore, among older adults, the ability to perform daily activities requiring lower limb strength differs between those with knee flexion–extension strength of 1.0–1.19 Nm/kg and those with ≥1.2 Nm/kg [8]. Based on these findings, we assumed a baseline value of 1.0 Nm/kg and post-intervention value of 1.2 Nm/kg and estimated the expected between-group difference after intake to be 0.2. With a planned sample size of 100 participants (50 per group) and assuming a standard deviation of 0.27, which is considered reasonable based on the variability reported in previous studies on knee extension strength in older adults [8], the 95% CI for the between-group difference was estimated to be 0.2 ± 0.114, yielding a coverage probability of 82%. This level of precision was considered sufficient compared with those used in previous studies. Therefore, the target sample size for this study was set at 100 participants, with 50 participants allocated to each group. The initial target sample size was 100 participants. However, recruitment was concluded with 50 participants because of several factors, including stringent eligibility criteria based on the AWGS 2019 sarcopenia diagnosis and unforeseen recruitment challenges arising from the COVID-19 pandemic.

### 2.4. Randomization and Blinding

Participants were randomly assigned (1:1) to the B-3HK or placebo group using a computer-generated randomization sequence stratified by sex and baseline grip strength. The allocation was concealed using an Interactive Web Response System (IWRS). The investigators, participants, and outcome assessors were blinded to group assignments throughout the study.

### 2.5. Intervention

The active product contained heat-inactivated, non-viable B-3HK at a dose equivalent to 2 × 10^10^ cells per day, administered orally once daily for 24 weeks. The placebo group received an identical capsule without B-3HK. Participants were instructed to maintain their usual diet and physical activity. Adherence was assessed by capsule counts at each study visit and calculated as the percentage of capsules consumed relative to the number prescribed.

### 2.6. Outcome Measures

The primary outcome was the change in knee extension strength, expressed in kilogram-force (kgf), from baseline to week 24, as measured using a standardized handheld dynamometer (μTas F-2; Anima Corporation, Tokyo, Japan). The secondary outcomes were SMI and limb muscle mass assessed using DXA and bioelectrical impedance analysis (BIA; MC-780MA-N; Tanita Corporation, Tokyo, Japan), bone mineral density, physical performance (the Short Physical Performance Battery (SPPB) score, gait speed, and grip strength), oral function (Eating Assessment Tool-10 (EAT-10) score), and exploratory serum biomarkers, including insulin-like growth factor-1 (IGF-1), 25-hydroxyvitamin D, C-reactive protein (CRP), and thyroid hormones. Safety outcomes included adverse events and routine hematological and biochemical laboratory assessments.

### 2.7. Gut Microbiota Analysis

Fecal samples were collected at baseline and week 24 for 16S rRNA sequencing. Bacterial DNA extraction and PCR amplification were performed following the established protocols [9]. The V3-V4 hypervariable regions of the 16S rRNA gene were amplified using region-specific primers and sequenced using a paired-end approach on the Illumina NextSeq 1000 platform using the NextSeq 1000/2000 P1 reagent kit (600 cycles) (Illumina, Inc., San Diego, CA, USA). The sequence data were analyzed using QIIME 2 version 2022.8 [10]. Demultiplexed reads were processed by filtering, denoising, merging, and chimera removal, and amplicon sequence variants were generated using DADA2 [11]. Taxonomic assignment was performed using the Greengenes2 database version 2022.10 [12]. Alpha diversity was presented as medians with interquartile ranges, and group differences were assessed using the Wilcoxon rank-sum test. Beta diversity was evaluated using Bray–Curtis, Jaccard, unweighted UniFrac, and weighted UniFrac distance metrics, followed by principal coordinate analysis (PCoA) visualization with 95% confidence ellipses. Group differences were evaluated using permutational multivariate analysis of variance with 999 permutations. Taxonomic composition at the phylum and genus levels was compared between groups separately at each time point using relative abundance. Only taxa with a median relative abundance greater than 1% in at least one group at either time point were included. Data were presented as medians with interquartile ranges. Group differences were analyzed using the Wilcoxon rank-sum test.

### 2.8. Statistical Analysis

Efficacy analyses were performed on the full analysis set (FAS), which followed the intention-to-treat principle and included all eligible randomized participants with evaluable efficacy data. Safety analyses were performed on the safety analysis set, which included all participants who received at least one dose of the study product. Continuous variables are presented as median (interquartile range [IQR]). Changes from baseline within each group were assessed using the Wilcoxon signed-rank test, and between-group differences in changes were evaluated using the Wilcoxon rank-sum test. For the primary endpoint, the between-group effect was additionally summarized as the Hodges–Lehmann estimate of the difference in change from baseline with a corresponding 95% confidence interval. No adjustment for multiple comparisons was applied to secondary or exploratory outcomes; all corresponding *p*-values are nominal and are intended for hypothesis generation rather than confirmatory inference. No imputation was applied for missing data, and the analyses were based on observed cases. Statistical analyses were conducted using SAS version 9.4 (SAS Institute Inc., Cary, NC, USA).

## 3. Results

### 3.1. Participants and Baseline Characteristics

**Figure 1:**
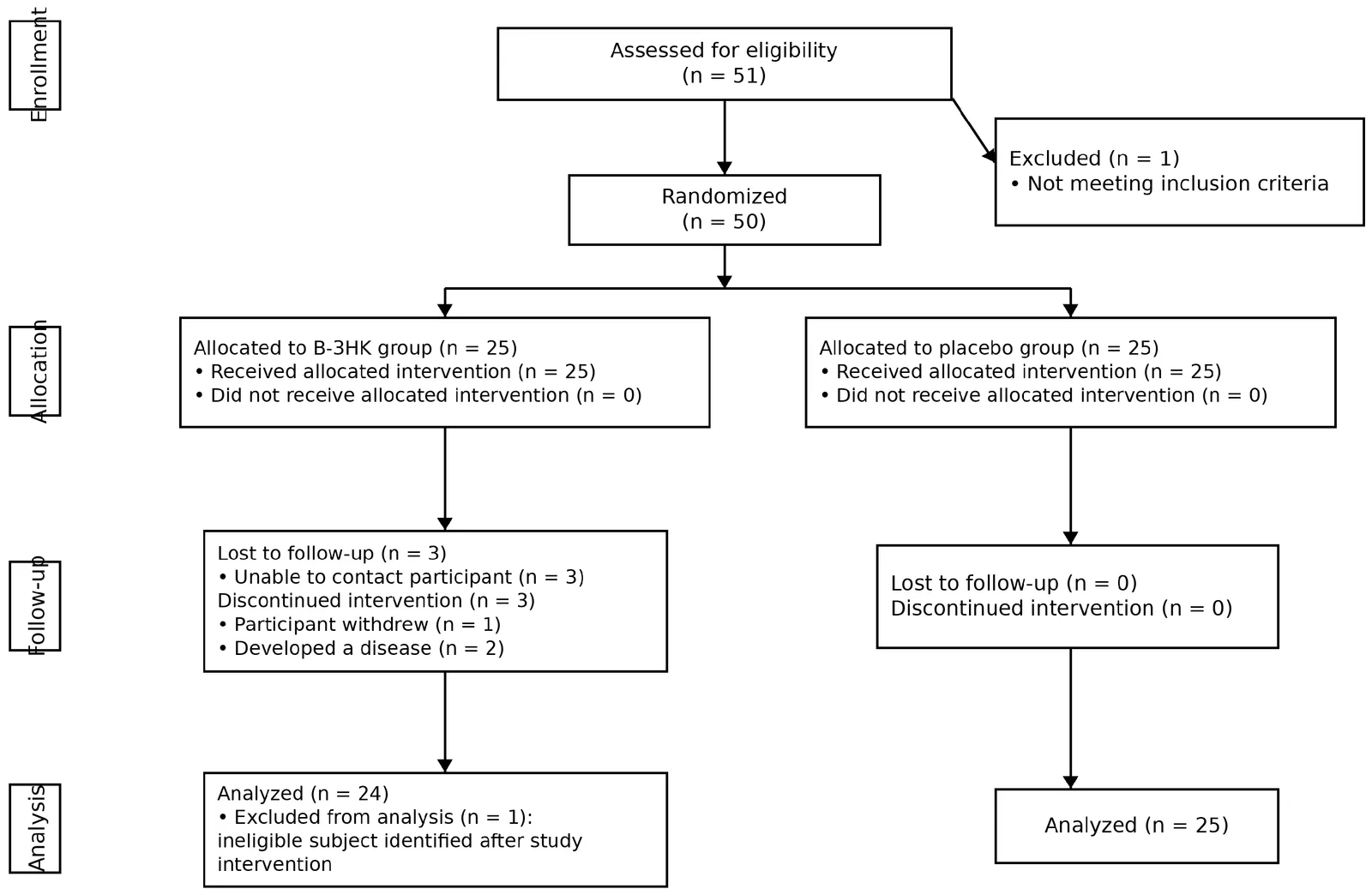
CONSORT flow diagram showing participant flow through the study.

Fifty participants were randomized to the B-3HK (*n* = 25) or placebo (*n* = 25) group (Figure 1). All the participants received the allocated intervention at baseline. One participant in the B-3HK group was excluded from the FAS because of ineligibility resulting from a deviation from the selection criteria, resulting in 24 participants for B-3HK and 25 participants for placebo. Consequently, the FAS consisted of 24 participants in the B-3HK group and 25 participants in the placebo group. The safety analysis set included all 50 randomized participants who received at least one dose of the study product. During follow-up, six participants in the B-3HK group were classified as lost to follow-up or had discontinued the intervention, whereas no participants in the placebo group were lost or had discontinued. Therefore, potential attrition should be considered when interpreting exploratory secondary outcomes. Adherence to the study product was high in both groups. The median adherence rate during the intervention period was 94.1% (IQR, 91.1–97.0%) in the B-3HK group and 94.1% (IQR, 93.5–100.0%) in the placebo group. The primary outcome analysis used a pre-specified FAS dataset. Baseline characteristics were similar between the groups (Table 1). The median age of the patients was approximately 80 years, and the sex distribution was balanced. Height, weight, body mass index, knee extension strength, and skeletal muscle indices exhibited no significant differences, except for normal walking speed, which was higher in the placebo group (*p* = 0.04).

### 3.2. Primary Outcome

Changes in knee extension strength over 24 weeks did not differ significantly between the groups (Table 2). No significant between-group differences were observed in changes in knee extension strength at week 12 (*p* = 0.656) or week 24 (*p* = 0.554). The Hodges–Lehmann estimate of the between-group difference in change from baseline was 0.40 kgf (95% CI, −1.30 to 2.20) at week 12 and 0.55 kgf (95% CI, −1.10 to 2.10) at week 24. The week-24 confidence interval included zero, providing no evidence of a between-group treatment effect on the primary endpoint.

### 3.3. Secondary Outcomes

**Figure 2:**
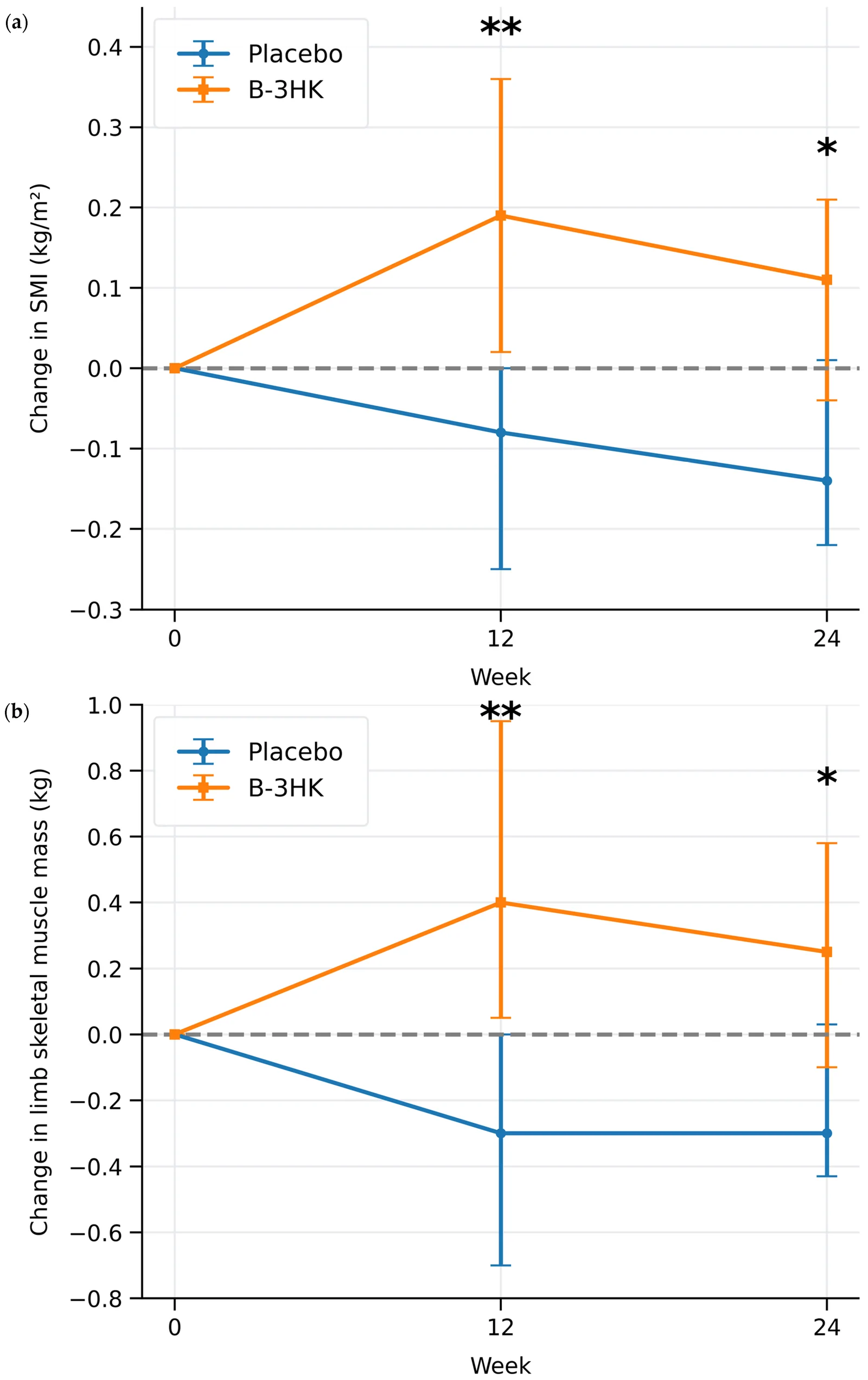
(**a**) Change in skeletal muscle mass index (SMI) and (**b**) limb skeletal muscle mass measured via bioelectrical impedance analysis (BIA) from baseline to weeks 12 and 24 in the placebo and B-3HK groups. Values are presented as median changes from baseline with interquartile ranges (IQRs). Week 0 was defined as baseline (Δ = 0). * nominal *p* < 0.05, ** nominal *p* < 0.01 between groups. The between-group differences should be interpreted as exploratory signals, primarily reflecting attenuation of the placebo-associated decline rather than a definitive absolute increase in the B-3HK group.

Among the exploratory secondary outcomes, muscle mass-related parameters assessed by bioelectrical impedance analysis (BIA) showed nominal between-group differences in the B-3HK group compared with those in the placebo group (Table 3, Figure 2). All *p*-values in this section are unadjusted nominal values and should not be interpreted as confirmatory evidence. At week 12, changes in SMI and limb skeletal muscle mass were nominally greater in the B-3HK group than in the placebo group (SMI: 0.19 [0.02, 0.36] vs. −0.08 [−0.25, 0.00] kg/m^2^, nominal *p* = 0.004; limb skeletal muscle mass: 0.40 [0.05, 0.95] vs. −0.30 [−0.70, 0.00] kg, nominal *p* = 0.003). These between-group differences persisted until week 24 (SMI, nominal *p* = 0.026; limb skeletal muscle mass, nominal *p* = 0.028). However, these signals were primarily driven by a decline in the placebo group, whereas within-group changes in the B-3HK group were not statistically significant. Accordingly, BIA findings are more appropriately interpreted as an attenuation of the placebo-associated decline in muscle mass-related indices rather than as definitive evidence of an absolute increase in muscle mass. No corresponding between-group differences were observed in grip strength, gait speed, SPPB, DXA-derived muscle indices, or phase angle (Table 3).

Regarding oral function, which is often impaired in patients with sarcopenia, EAT-10 scores exhibited a nominally significant exploratory improvement in the B-3HK group compared with that in the placebo group at week 24 (−0.5 [−2.0, 0.0] vs. 0.0 [0.0, 0.0]; nominal *p* = 0.013) (Table 3). However, the baseline EAT-10 scores were low in both groups and below the commonly used screening threshold, indicating that this change occurred in participants with minimal swallowing-symptom burden. By contrast, the oral frailty scores did not show significant changes in either group.

DXA-derived skeletal muscle mass indices showed no significant between-group differences and are presented together with the BIA indices in Table 3. DXA-derived bone parameters and regional non-fat mass did not differ between groups, as presented in Table S1.

**Figure 3:**
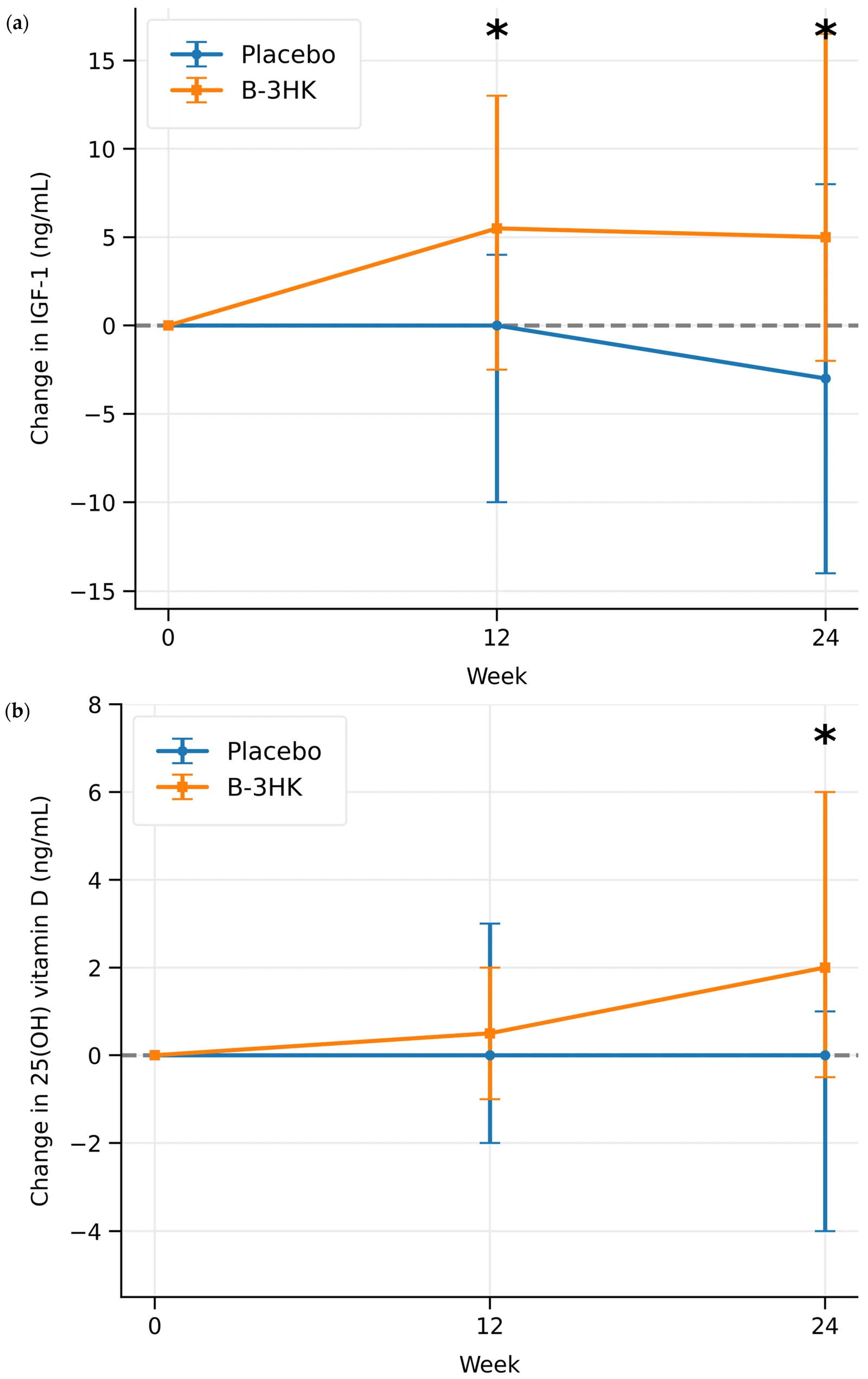
Change in serum (**a**) insulin-like growth factor-1 (IGF-1) and (**b**) 25-hydroxyvitamin D levels from baseline to weeks 12 and 24 in the placebo and B-3HK groups. Values are presented as median changes from baseline with interquartile ranges (IQRs). Week 0 was defined as baseline (Δ = 0). * nominal *p* < 0.05, between groups.

Serum IGF-1 levels exhibited nominal increases in the B-3HK group compared with that in the placebo group at both week 12 (5.5 [−2.5, 13.0] vs. 0.0 [−10.0, 4.0] ng/mL; nominal *p* = 0.016) and week 24 (5.0 [−2.0, 16.5] vs. −3.0 [−14.0, 8.0] ng/mL; nominal *p* = 0.036). This signal was supported by a within-group increase in the B-3HK group and was the most biologically coherent exploratory endocrine finding. Serum 25(OH) vitamin D also exhibited a nominal increase in the B-3HK group at week 24 (2.0 [−0.5, 6.0] vs. 0.0 [−4.0, 1.0] ng/mL; nominal *p* = 0.013) (Table 3, Figure 3).

### 3.4. Gut Microbiota Composition

Gut microbiota composition was evaluated using 16S rRNA gene sequencing of fecal samples collected at baseline and week 24. Alpha diversity indices, including observed amplicon sequence variants, Shannon index, and Faith’s phylogenetic diversity, did not differ significantly between the B-3HK and placebo groups at baseline and exhibited no significant within- or between-group changes over the 24-week intervention period (Table S2).

Beta diversity analysis demonstrated no distinct clustering in the treatment group at either baseline or week 24, as visualized by PCoA (Figure S1). Permutational multivariate analysis of variance confirmed the absence of a significant effect of B-3HK intake on the overall microbial community structure.

At the taxonomic level, the relative abundances of the dominant bacterial phyla and genera remained comparable between the groups throughout the study period, and no taxa exhibited significant differential changes associated with the intervention (Table S3).

### 3.5. Safety

No serious adverse events occurred during the 24-week intervention period. Minor adverse events, such as transient gastrointestinal symptoms (e.g., mild constipation or diarrhea), were observed in both groups without significant between-group differences. All adverse events were resolved spontaneously and considered unrelated to the study product. A modest but statistically significant reduction in platelet counts was observed in the B-3HK group at weeks 12 and 24 compared with that in the placebo group; however, all values remained within the normal reference range, and no bleeding events or clinically relevant hematological abnormalities were observed (Table S1). This platelet change was recorded as a safety-related hematological finding and may also represent an exploratory biological signal potentially related to host immune or inflammatory regulation, although this interpretation remains speculative and requires confirmation in future studies. No clinically relevant changes were detected in the other hematological, hepatic, renal, or biochemical parameters.

## 4. Discussion

In this 24-week, randomized, placebo-controlled pilot trial, B-3HK did not significantly improve the primary outcome of knee extension strength in patients with sarcopenia. Exploratory analyses identified nominal between-group differences in selected body composition, swallowing-related symptoms, and endocrine outcomes. However, these findings were not adjusted for multiplicity, were not supported by corresponding functional outcomes, and therefore should be regarded as hypothesis-generating rather than confirmatory evidence of efficacy. Nevertheless, these exploratory signals may help inform the selection of mechanistic and clinical endpoints for future adequately powered trials.

The absence of a significant effect on knee extension strength was a critical finding. Muscle strength is a multifactorial outcome influenced by muscle mass, as well as by neuromuscular coordination, motor unit recruitment, and habitual physical activity. In patients with established sarcopenia, age-related anabolic resistance and impaired neuromuscular adaptability may limit the translation of biological or endocrine signals for measurable functional improvement. In addition, the present intervention was not combined with structured resistance training, which is known to synergize with anabolic signaling pathways, such as Akt-mTOR signaling. Therefore, the lack of improvement in the primary outcome does not necessarily negate the biological activity of the intervention but suggests that B-3HK alone is insufficient to induce clinically meaningful gains in muscle strength within 24 weeks in this study.

An important observation in this trial was the discrepancy between the muscle mass assessments conducted using BIA and DXA. Although BIA-derived indices exhibited nominal between-group differences favoring B-3HK, DXA-derived indices did not. Moreover, the BIA signal primarily reflected the attenuation of the decline observed in the placebo group rather than a robust within-group increase in the B-3HK group. This suggests that B-3HK may exert a stabilizing effect on muscle-related parameters rather than promoting overt hypertrophy. This effect is biologically plausible, particularly in populations characterized by progressive muscle decline. The discrepancy between modalities likely reflects differences in measurement sensitivity, with BIA potentially capturing early changes in soft-tissue properties, intracellular hydration, or muscle quality, whereas DXA primarily reflects mineral-free lean mass and may be less sensitive to subtle or short-term changes. These findings support the interpretation that B-3HK may influence early or functional components of body composition, although definitive evidence for muscle mass gain has not been established.

Importantly, the gut microbiota composition remained substantially unchanged following B-3HK intake, with no significant differences in alpha diversity, beta diversity, or taxonomic profiles between the intervention and placebo groups. This finding is notable because previous probiotic interventions targeting muscle-related outcomes have often been accompanied by detectable shifts in the microbial composition or diversity. However, in the present study, the absence of compositional changes indicated that the observed endocrine- and body composition-related signals were not mediated by alterations in the global structure of the gut microbiota. This null finding is biologically plausible, considering the non-viable nature of the intervention. Because B-3HK is heat-killed, it does not colonize the intestinal tract or contribute directly to microbial metabolic activities, such as short-chain fatty acid production. Accumulating evidence has shown that non-viable microbial components can exert biological effects through host-mediated pathways, particularly via pattern-recognition receptor signaling in immune cells. Therefore, the lack of microbiota compositional shifts in the present trial supports the interpretation that B-3HK may act, at least in part, through host immune–endocrine interactions rather than through microbiota remodeling. Importantly, 16S rRNA gene sequencing provides information on microbial composition, but not on microbial function or host–microbe signaling dynamics. Subtle functional effects, including changes in microbial gene expression, metabolite signaling, or mucosal immune responses, may not be captured by community-level compositional analyses. Thus, although B-3HK intake did not induce overt changes in the gut microbiota structure, functional host responses independent of detectable microbiota shifts could not be excluded.

Among the secondary outcomes, an increase in serum IGF-1 levels was noteworthy. IGF-1 is a central anabolic regulator of skeletal muscle protein synthesis via the Akt-mTOR pathway [13]. The observed increase, supported by within-group changes in the B-3HK group, provides biologically plausible evidence that B-3HK influences host anabolic signaling. However, the lack of a direct association with skeletal muscle mass highlights the complexity of anabolic resistance in sarcopenia and suggests that endocrine modulation alone may be insufficient to induce measurable hypertrophy in the absence of concurrent resistance training. Age-related anabolic resistance may further blunt the translation of endocrine signals into measurable muscle mass gains in the absence of concurrent resistance training [13,14]. A plausible explanation for the dissociation between increased circulating IGF-1 levels and lack of a proportional increase in skeletal muscle mass is the regulatory role of insulin-like growth factor-binding proteins (IGFBPs). Most circulating IGF-1 is bound to IGFBPs, and effective tissue action depends on total IGF-1, as well as on IGF bioavailability, complex formation, and tissue-specific IGFBP expression and processing. IGFBP-3 is a major carrier of circulating IGF-1, whereas IGFBP-5 is highly expressed in skeletal muscles and may modulate local IGF signaling via autocrine and paracrine mechanisms [15,16,17]. However, because IGFBPs and bioavailable IGF-1 were not assessed in the present study, these mechanistic interpretations remain speculative. Given that B-3HK is heat-killed, the canonical gut microbiota–SCFA axis is unlikely to explain the observed endocrine changes. Rather, accumulating experimental evidence shows that host IGF-1 levels can be modulated via immune-mediated pathways independent of microbiota compositional shifts [18]. Heat-killed lactic acid bacteria exert immunomodulatory effects via pattern recognition receptor signaling even in the absence of viable bacteria [19,20]. Such immune activation may secondarily influence hepatic endocrine outputs, including IGF-1 and systemic inflammatory tone, which are relevant to muscle remodeling [21,22].

In parallel, the serum 25-hydroxyvitamin D levels exhibited a nominal increase in the B-3HK group. Previous meta-analyses have reported the heterogeneous effects of vitamin D supplementation on muscle outcomes, with small or inconsistent effects on muscle mass and strength [23]. The present findings highlight the possibility that B-3HK-associated host responses may secondarily influence vitamin D status rather than reflecting a direct microbiota-driven mechanism. However, this interpretation remains exploratory because vitamin D intake, sunlight exposure, and seasonal effects were not fully addressed in the present analysis.

The exploratory reduction in EAT-10 scores may also be related to mucosal immune modulation. Prior studies have shown that both viable and heat-killed probiotic preparations can increase salivary IgA levels and anti-inflammatory cytokines such as interleukin-10 and transforming growth factor-β, which are mechanistically consistent with the observed improvements in swallowing-related symptoms [24]. However, because baseline EAT-10 scores were low, the clinical magnitude of this change should be interpreted with caution.

The modest decrease in platelet count observed in the B-3HK group also deserves careful interpretation. As the values remained within the normal reference range and were not accompanied by bleeding or clinically relevant hematological abnormalities, this finding does not appear to indicate safety concerns in the present pilot trial. Platelets are increasingly recognized as mediators of inflammation and immune regulation, and chronic inflammatory states are associated with platelet production or activation. Therefore, a decrease in platelet count may reflect an immunomodulatory or anti-inflammatory host response to B-3HK. However, because conventional inflammatory markers, such as CRP and IL-6, did not exhibit corresponding nominal improvements, this interpretation remains speculative and hypothesis-generating.

This study has several limitations. The study was substantially under-recruited relative to the planned sample size of 100 participants, resulting in limited precision and reduced ability to detect clinically meaningful treatment effects. Recruitment was completed for 50 participants despite an initial target sample size of 100, and discontinuation/loss to follow-up occurred only in the B-3HK group. Therefore, attrition bias cannot be excluded. The intervention period was limited to 24 weeks and may have been insufficient to detect clinically meaningful changes in muscle strength or mass; moreover, the intervention did not include structured resistance training, which is known to synergize with endocrine signals for hypertrophy in patients with sarcopenia. Secondary outcomes were numerous and were not adjusted for multiplicity; therefore, all secondary *p*-values should be interpreted as nominal and hypothesis-generating. The discrepancy between BIA and DXA findings, low baseline EAT-10 scores, and lack of clear corresponding changes in CRP or IL-6 further limited the mechanistic interpretation. Importantly, IGF-binding proteins were not measured; therefore, we could not determine whether changes in IGFBP profiles mediated the relationship between increased circulating IGF-1 levels and muscle mass outcomes. Future studies on measurements of IGFBPs, bioavailable IGF-1, inflammatory and immune markers, and prespecified sensitivity analyses are essential to clarify these mechanisms.

As a pilot study, these data may inform planning of future efficacy trials. Using week-24 primary-endpoint data and the method of Wan et al. [25] to approximate means and standard deviations from medians and interquartile ranges yielded a standardized effect size of approximately 0.30. Under conventional assumptions of a two-sided α = 0.05, 80% power, and equal allocation, a future trial would require approximately 175–180 evaluable participants per group. This estimate is provisional because effect size estimates from small pilot samples are imprecise, and a confirmatory trial should prespecify a clinically meaningful target difference and inflate recruitment for expected attrition.

## 5. Conclusions

B-3HK did not improve knee extension strength, the primary endpoint in patients with sarcopenia; therefore, clinical efficacy was not demonstrated in this pilot trial. The nominal differences in secondary body-composition and endocrine outcomes were derived from multiple unadjusted exploratory comparisons and should be regarded only as hypothesis-generating. These data may inform the design and mechanistic endpoints of larger, adequately powered trials, but confirmation is required before any therapeutic benefit can be inferred.

## Figures and Tables

**Table 1:** Baseline characteristics of participants in the placebo and B-3HK groups.

Characteristic	Placebo (N = 25)	B-3HK (N = 24)	*p*-Value
Age	81 (75, 84)	80 (74, 84)	0.869
Sex (Male/Female)	13/12	13/11	0.889
Height (cm)	154.0 (149.6, 162.5)	156.8 (151.4, 161.8)	0.866
Body weight (kg)	52.2 (44.0, 59.0)	49.8 (44.1, 53.5)	0.104
BMI	21.0 (19.0, 23.0)	20.2 (18.7, 21.7)	0.123
Knee extension strength (kgf)	22.3 (13.2, 24.3)	18.6 (13.7, 22.8)	0.624
SMI by BIA (kg/m^2^)	5.95 (5.67, 6.74)	5.85 (5.44, 6.12)	0.112
SMI by DXA (kg/m^2^)	5.35 (5.16, 6.34)	5.40 (5.05, 5.62)	0.447
Grip strength (kg)	20.0 (14.0, 22.0)	14.8 (13.0, 20.6)	0.331
Walking speed (m/s)	1.04 (0.88, 1.38)	0.96 (0.76, 1.14)	0.04 *

Data are presented as median (interquartile range). Statistical significance is indicated as follows: * *p* < 0.05, Wilcoxon rank-sum test between groups.BMI, body mass index; SMI, skeletal muscle mass index; BIA, bioelectrical impedance analysis; DXA, dual-energy X-ray absorptiometry.

**Table 2:** Changes in knee extension strength at weeks 12 and 24.

Variable	Week	N (P)	N (B)	Placebo	B-3HK	Within *p* (P)	Within *p* (B)	Δ Placebo	Δ B-3HK	HL Estimate (95% CI)	Between *p*
Knee extension strength (kgf)	week 0	25	24	22.3 (13.2, 24.3)	18.6 (13.7, 22.8)	—	—	—	—	—	—
	week 12	25	20	22.0 (13.3, 23.6)	18.7 (14.2, 20.1)	1	0.538	−0.1 (−1.1, 1.3)	0.6 (−1.1, 2.1)	0.40 (−1.30 to 2.20)	0.656
	week 24	25	18	20.1 (12.9, 25.2)	17.0 (14.4, 20.0)	0.6	0.514	0.1 (−2.9, 1.6)	0.5 (−1.1, 1.7)	0.55 (−1.10 to 2.10)	0.554

Data are presented as median (interquartile range). N indicates the number of participants with evaluable data at each time point and may vary because of missing observations, discontinuation, or loss to follow-up. Changes (Δ) represent changes from baseline. The between-group effect is presented as the Hodges–Lehmann estimate of the difference in change from baseline with its corresponding 95% confidence interval. Between-group differences were assessed using the Wilcoxon rank-sum test, and within-group changes were assessed using the Wilcoxon signed-rank test. P, placebo; B, B-3HK; HL, Hodges–Lehmann; CI, confidence interval.

**Table 3:** Secondary outcomes: body composition (BIA and DXA), physical performance, and endocrine and exploratory markers.

Variable	Week	N (Placebo)	N (B-3HK)	Placebo	B-3HK	Within-Group *p*-Value (Placebo)	Within-Group *p*-Value (B-3HK)	Δ Placebo	Δ B-3HK	Between-Groups *p*-Value
Body Composition										
SMI (kg/m^2^), BIA method	week 0	24	24	5.95 (5.67, 6.74)	5.85 (5.44, 6.12)	—	—	—	—	—
	week 12	24	20	5.90 (5.61, 6.82)	5.98 (5.67, 6.52)	0.010 *	0.177	−0.08 (−0.25, 0.00)	0.19 (0.02, 0.36)	0.004 **
	week 24	24	18	6.07 (5.63, 6.60)	5.76 (5.47, 6.10)	0.039 *	0.266	−0.14 (−0.22, 0.01)	0.11 (−0.04, 0.21)	0.026 *
Limb skeletal muscle mass (kg), BIA method	week 0	24	24	14.35 (12.88, 16.35)	14.10 (12.75, 15.88)	—	—	—	—	—
	week 12	24	20	13.70 (12.80, 16.28)	14.85 (12.20, 15.90)	0.005 **	0.173	−0.30 (−0.70, 0.00)	0.40 (0.05, 0.95)	0.003 **
	week 24	24	18	14.40 (12.60, 16.33)	14.00 (12.33, 15.88)	0.035 *	0.231	−0.30 (−0.43, 0.03)	0.25 (−0.10, 0.58)	0.028 *
SMI (kg/m^2^), DXA method	week 0	25	24	5.35 (5.16, 6.34)	5.40 (5.05, 5.62)	—	—	—	—	—
	week 24	25	18	5.43 (4.96, 6.44)	5.47 (5.12, 5.82)	0.936	0.156	0.01 (−0.16, 0.17)	0.06 (−0.07, 0.23)	0.242
Limb skeletal muscle mass (kg), DXA method	week 0	25	24	13.05 (11.58, 15.90)	13.39 (11.25, 14.67)	—	—	—	—	—
	week 24	25	18	13.10 (11.24, 15.96)	12.72 (11.35, 15.18)	0.893	0.154	0.01 (−0.47, 0.40)	0.15 (−0.14, 0.51)	0.242
Phase angle, BIA method	week 0	24	24	4.35 (3.88, 4.80)	3.85 (3.40, 4.50)	—	—	—	—	—
	week 12	24	20	4.25 (4.00, 4.53)	4.15 (3.60, 4.63)	0.545	0.636	0.00 (−0.20, 0.20)	0.05 (−0.20, 0.23)	0.545
	week 24	24	18	4.25 (3.88, 4.63)	4.00 (3.60, 4.45)	0.274	0.552	0.00 (−0.23, 0.10)	0.10 (−0.18, 0.30)	0.277
Physical performance										
Grip strength (kg)	week 0	25	24	20.0 (14.0, 22.0)	14.8 (13.0, 20.6)	—	—	—	—	—
	week 12	25	20	19.0 (16.0, 24.0)	15.3 (12.9, 20.3)	0.374	0.84	1.0 (−1.0, 2.0)	0.3 (−1.1, 1.6)	0.614
	week 24	25	18	19.0 (16.0, 22.0)	15.3 (13.3, 19.5)	0.693	0.232	0.0 (−1.5, 1.0)	−0.5 (−2.0, 1.0)	0.568
Gait speed (m/s)	week 0	25	24	1.04 (0.88, 1.38)	0.96 (0.76, 1.14)	—	—	—	—	—
	week 12	25	20	1.14 (0.99, 1.32)	0.96 (0.75, 1.09)	0.742	0.231	0.01 (−0.07, 0.07)	−0.02 (−0.08, 0.02)	0.235
	week 24	25	18	1.06 (0.92, 1.35)	0.79 (0.73, 1.08)	1	0.472	0.05 (−0.16, 0.12)	−0.02 (−0.20, 0.10)	0.571
SPPB (score)	week 0	25	24	11.0 (9.0, 12.0)	10.0 (8.8, 12.0)	—	—	—	—	—
	week 12	25	20	11.0 (9.0, 12.0)	11.0 (7.0, 12.0)	0.599	0.615	0.0 (0.0, 1.0)	0.0 (0.0, 0.3)	0.97
	week 24	25	18	11.0 (9.0, 12.0)	9.5 (8.0, 10.8)	0.968	0.173	0.0 (0.0, 1.0)	−0.5 (−1.0, 0.0)	0.141
Other exploratory markers										
EAT-10 (deglutition screening tool)	week 0	25	24	0 (0, 1)	0 (0, 1.3)	—	—	—	—	—
	week 12	25	21	0 (0, 0)	0 (0, 0)	0.386	0.082	0 (0, 0)	0 (−2, 0)	0.076
	week 24	25	18	0 (0, 1)	0 (0, 0)	0.901	0.009 **	0 (0, 0)	−0.5 (−2, 0)	0.013 *
IGF-1 (ng/mL)	week 0	25	25	70.0 (59.0, 82.0)	72.0 (59.0, 96.0)	—	—	—	—	—
	week 12	25	22	68.0 (54.0, 82.0)	73.0 (61.5, 92.3)	0.254	0.021 *	0.0 (−10.0, 4.0)	5.5 (−2.5, 13.0)	0.016 *
	week 24	25	19	67.0 (54.0, 83.0)	81.0 (64.0, 101.5)	0.353	0.045 *	−3.0 (−14.0, 8.0)	5.0 (−2.0, 16.5)	0.036 *
25(OH) vitamin D (ng/mL)	week 0	25	25	18.0 (15.0, 25.0)	21.0 (12.0, 23.0)	—	—	—	—	—
	week 12	25	22	20.0 (14.0, 24.0)	19.0 (15.3, 25.0)	0.443	0.36	0.0 (−2.0, 3.0)	0.5 (−1.0, 2.0)	0.838
	week 24	25	19	16.0 (11.0, 24.0)	23.0 (20.5, 27.5)	0.287	0.023 *	0.0 (−4.0, 1.0)	2.0 (−0.5, 6.0)	0.013 *

Data are presented as median (interquartile range). N indicates the number of participants with evaluable data for each outcome at each time point and may vary because of outcome-specific missing data, discontinuation, or loss to follow-up. Exploratory biomarker variables were summarized using all available measurements. Statistical significance is indicated as follows: * nominal *p* < 0.05; ** nominal *p* < 0.01, Wilcoxon rank-sum test between groups, and Wilcoxon signed-rank test within groups. Body composition (BIA and DXA), physical performance, endocrine, and other key exploratory outcomes are presented in this table; remaining bone, gastrointestinal, safety-related laboratory, and hematological indices, together with regional DXA non-fat mass, thyroid hormones, testosterone, CRP, and IL-6, are presented in Table S1. No adjustment for multiple comparisons was performed. All secondary *p*-values are nominal and exploratory, and placement of variables in Table 3 does not imply confirmatory status; remaining exploratory outcomes are reported in Table S1.

## Data Availability

The data presented in this study are available from the corresponding author upon reasonable request and are subject to ethical and privacy restrictions.

## Supplementary Materials

The following supporting information can be downloaded at: https://www.mdpi.com/article/10.3390/nu18183074/s1, Figure S1: Principal coordinate analysis (PCoA) of fecal microbiota beta diversity based on Bray–Curtis, Jaccard, unweighted UniFrac, and weighted UniFrac distances in the placebo and B-3HK groups at week 0 and week 24; Table S1: Additional exploratory, safety-related laboratory, and hematological outcomes in the placebo and B-3HK groups; Table S2: Alpha diversity of fecal microbiota in the placebo and B-3HK groups; Table S3: Composition of the fecal microbiota at the phylum and genus levels in the placebo and B-3HK groups.

## Abbreviations

The following abbreviations are used in this manuscript: AWGS—Asian Working Group for Sarcopenia; B-3HK—heat-killed *Bifidobacterium breve* B-3; BIA—bioelectrical impedance analysis; DXA—dual-energy X-ray absorptiometry; EAT-10—Eating Assessment Tool-10; FAS—full analysis set; IGF-1—insulin-like growth factor-1; SMI—skeletal muscle mass index; SPPB—Short Physical Performance Battery.
