# Supplementary material for: Effects of Heat-Killed Bifidobacterium breve B-3, a Postbiotic, on Muscle Mass-Related Parameters in Patients with Sarcopenia: A Randomized, Double-Blind, Placebo-Controlled Pilot Trial
Source: Nutrients. 2026 Sep 20;18(18):3074. doi: 10.3390/nu18183074 (PMC13610449; doi:10.3390/nu18183074)
Supplement: Supplementary file 1 [file nutrients-18-03074-s001.zip › Table S1.pdf]

Table S1. Additional exploratory, safety-related laboratory, and hematological outcomes in the placebo and B-3HK groups.

		Week	N (Placebo)	N (B-3HK)	Placebo	B-3HK		Within-group p-value			Difference between changes from week 0		
								Placebo	B-3HK		Placebo	B-3HK	Between-groups p-value
Non-fat mass (g), DXA method	both arms	week 0	25	24	3191 (2612, 3944)	2869 (2571, 3615)		-	-		-	-	-
		week 24	25	18	3236 (2575, 3904)	2873 (2661, 3536)		0.201	0.601		-36 (-227, 96)	-12 (-96, 122)	0.214
	both legs	week 0	25	24	9857 (9232, 11676)	10159 (8764, 11465)		-	-		-	-	-
		week 24	25	18	9978 (8665, 11801)	9617 (8844, 11812)		0.474	0.369		225 (-296, 326)	213 (-192, 448)	0.605
Lumbar vertebrae	BMD (g/cm²)	week 0	25	24	1.057 (0.879, 1.223)	1.036 (0.896, 1.369)		-	-		-	-	-
		week 24	25	18	1.071 (0.845, 1.218)	0.952 (0.875, 1.347)		0.419	0.446		-0.003 (-0.030, 0.015)	0.003 (-0.019, 0.034)	0.242
	T-score	week 0	25	24	-0.7 (-1.9,	-0.8 (-1.9,		-	-		-	-	-

					0.5)	1.4)							
		week 24	25	18	-0.6 (-2.1, 0.4)	-1.2 (-2.2, 1.3)		0.701	0.312		0.0 (-0.2, 0.1)	0.05 (-0.1, 0.3)	0.242
	Z-score	week 0	25	24	1.2 (0.4, 2.1)	1.3 (0.9, 2.8)		-	-		-	-	-
		week 24	25	18	1.2 (0.6, 2.2)	1.1 (0.5, 3.0)		0.807	0.164		0.0 (-0.1, 0.01)	0.05 (-0.1, 0.3)	0.233
	YAM (%)	week 0	25	24	91.1 (74.2, 107.5)	89.7 (76.0, 120.3)		-	-		-	-	-
		week 24	25	18	89.9 (70.9, 102.2)	79.9 (73.4, 113.0)		0.003**	0.199		-2.8 (-5.8, -0.1)	-1.6 (-3.3, 1.1)	0.192
Femoral neck	BMD (g/cm²)	week 0	24	24	0.738 (0.646, 0.812)	0.730 (0.591, 0.798)		-	-		-	-	-
		week 24	24	18	0.745 (0.673, 0.798)	0.730 (0.572, 0.803)		0.867	0.879		-0.002 (-0.014, 0.019)	0.000 (-0.024, 0.032)	0.919
	T-score	week 0	24	24	-2.0 (-2.7, -1.3)	-2.1 (-3.0, -1.7)		-	-		-	-	-
		week 24	24	18	-2.1 (-2.7, -1.6)	-2.2 (-3.2, -1.7)		0.856	1.000		0.0 (-0.1, 0.2)	0.05 (-0.2, 0.3)	1.000
	Z-score	week 0	24	24	0.4 (-0.3, 1.0)	0.3 (-0.4, 0.5)		-	-		-	-	-
		week 24	24	18	0.2 (-0.4,	0.2 (-0.4,		0.486	0.678		0.0 (-0.1,	0.05 (-0.2,	0.990

					0.8)	0.6)					0.2)	0.3)	
	YAM (%)	week 0	24	24	75.6 (67.6, 83.9)	74.9 (62.9, 79.9)		-	-		-	-	-
		week 24	24	18	79.3 (71.7, 85.0)	77.7 (60.9, 85.5)		0.02*	0.016*		3.9 (-0.4, 6.7)	4.3 (-0.1, 6.7)	1.000
Total hip	BMD (g/cm²)	week 0	24	24	0.804 (0.713, 0.852)	0.788 (0.618, 0.853)		-	-		-	-	-
		week 24	24	18	0.780 (0.685, 0.848)	0.774 (0.614, 0.816)		0.808	0.862		-0.001 (-0.016, 0.001)	-0.005 (-0.013, 0.016)	0.980
	T-score	week 0	24	24	-1.8 (-2.4, -1.1)	-1.8 (-2.6, -1.3)		-	-		-	-	-
		week 24	24	18	-1.9 (-2.3, -1.4)	-2.0 (-2.7, -1.6)		0.776	0.841		0.0 (-0.1, 0.1)	-0.05 (-0.1, 0.1)	1.000
	Z-score	week 0	24	24	0.2 (-0.3, 0.8)	0.2 (-0.4, 0.7)		-	-		-	-	-
		week 24	24	18	0.0 (-0.3, 0.6)	0.3 (-0.3, 0.6)		0.865	0.401		0.0 (-0.1, 0.1)	0.0 (-0.1, 0.1)	0.550
	YAM (%)	week 0	24	24	77.0 (69.3, 86.0)	76.4 (64.3, 83.1)		-	-		-	-	-
		week 24	24	18	81.1 (71.2, 88.3)	80.5 (63.8, 84.9)		0.005**	0.001**		3.3 (-0.1, 7.3)	4.8 (0.4, 6.3)	0.909

Oral frailty (score)		week 0	25	24	3.0 (2.0, 4.0)	3.5 (2.0, 6.3)		-	-		-	-	-
		week 12	25	21	3.0 (2.0, 4.0)	4.0 (2.0, 6.0)		0.904	0.860		0.0 (-1.0, 1.0)	0.0 (-1.0, 1.0)	0.857
		week 24	25	18	3.0 (2.0, 4.0)	3.5 (3.0, 5.8)		0.791	0.832		0.0 (-2.0, 1.0)	0.0 (-1.8, 0.8)	0.774
Izumo scale (score)	Heartburn	week 0	25	24	0 (0, 1)	0 (0, 0)		-	-		-	-	-
		week 12	25	20	0 (0, 2)	0 (0, 2)		0.163	0.382		0 (0, 2)	0 (0, 1.3)	0.885
		week 24	25	17	0 (0, 1)	0 (0, 2)		0.918	0.725		0 (0, 0)	0 (0, 1)	0.589
	stomach pain	week 0	25	24	0 (0, 0)	0 (0, 0)		-	-		-	-	-
		week 12	25	20	0 (0, 2)	0 (0, 2)		0.798	0.433		0 (0, 0)	0 (0, 2)	0.566
		week 24	25	18	0 (0, 0)	0 (0, 0)		0.398	0.892		0 (0, 0)	0 (0, 0)	0.844
	heavy stomach	week 0	25	24	0 (0, 3)	1 (0, 2)		-	-		-	-	-
		week 12	25	20	1 (0, 2)	0.5 (0, 3)		0.177	0.892		0 (-2, 0)	0 (-1, 0.3)	0.567
		week 24	25	18	0 (0, 1)	0 (0, 1.8)		0.103	0.904		0 (-2, 0)	0 (-1, 0)	0.642
	constipation	week 0	25	24	1 (0, 4)	0.5 (0, 5.5)		-	-		-	-	-
		week 12	25	20	0 (0, 1)	1.5 (0, 3.5)		0.037*	0.916		0 (-2, 0)	0 (-2, 1.3)	0.369
		week 24	25	18	1 (0, 3)	1 (0, 2)		0.612	0.287		0 (-1, 1)	0 (-2, 0.8)	0.567
	diarrhea	week 0	25	24	0 (0, 4)	0 (0, 2)		-	-		-	-	-
		week 12	25	20	0 (0, 1)	1 (0, 2.3)		0.152	0.944		0 (-1, 0)	0 (-0.5, 1)	0.218
		week 24	25	18	0 (0, 2)	0 (0, 0.8)		0.270	0.263		0 (-2, 0)	0 (-0.8, 0)	0.968
Modified constipation scoring system (M-CSS)		week 0	25	24	2 (0, 4)	2.5 (1, 4.3)		-	-		-	-	-
		week 12	25	21	2 (0, 4)	2 (1, 4)		0.407	0.216		0 (-2, 1)	0 (-2, 0)	0.640
		week 24	25	18	3 (1, 6)	2 (1, 3)		0.868	0.037*		0 (-1, 1)	-1 (-2, 0)	0.052

(score)												
constipation scoring system (CSS) (score)	week 0	25	24	2 (0, 8)	3 (1, 6)		-	-		-	-	-
	week 12	25	21	2 (0, 7)	2 (1, 5)		0.483	0.205		0 (-2, 1)	0 (-2, 0)	0.579
	week 24	25	18	4 (1, 7)	2 (1, 4)		0.876	0.095		0 (-1, 1)	-1 (-2, -0.3)	0.053
DVS（Total score 10days）(score)	week 0	24	23	65.0 (48.0, 72.3)	66.0 (54.5, 77.0)		-	-		-	-	-
	week 12	24	20	63.0 (44.0, 74.8)	65.0 (47.5, 78.5)		0.702	0.521		0.0 (-5.3, 6.3)	-0.5 (-2.5, 2.3)	0.464
	week 24	24	17	61.5 (46.5, 72.0)	62.0 (57.0, 78.0)		0.920	0.826		0.0 (-4.0, 2.8)	0.0 (-3.0, 6.0)	1.000
platelet (10^9/L)	week 0	25	25	194 (164, 230)	244 (183, 263)		-	-		-	-	-
	week 12	25	22	202 (172, 262)	224 (175, 255)		0.016*	0.404		10 (-3, 31)	-5.5 (-19, 9)	0.036*
	week 24	25	19	206 (181, 235)	208 (162, 237)		0.005**	0.049**		7 (4, 31)	-14 (-28, -3)	0.001**
AST(GOT) (U/L)	week 0	25	25	23 (20, 26)	21 (17, 25)		-	-		-	-	-
	week 12	25	22	24 (20, 29)	21 (18, 24)		0.836	0.294		0 (-1, 2)	-1 (-3, 1)	0.428
	week 24	25	19	25 (20, 26)	22 (20, 26)		0.568	0.417		1 (-2, 3)	1 (-1, 3)	0.886
ALT(GPT) (U/L)	week 0	25	25	17 (14, 20)	13 (9, 14)		-	-		-	-	-
	week 12	25	22	15 (13, 19)	12 (10, 14)		0.482	0.535		-1 (-4, 2)	1 (-1, 3)	0.397
	week 24	25	19	15 (13,17)	12 (9, 16)		0.292	0.285		0 (-4, 2）	1 (-1, 2)	0.220
ALP (U/L)	week 0	25	25	90 (66, 105)	73 (58, 83)		-	-		-	-	-

	week 12	25	22	88 (63, 95)	68 (56, 82)		0.054	0.917		-5 (-15, 3)	1 (-5, 6)	0.112
	week 24	25	19	90 (65, 99)	68 (59, 84)		0.345	0.825		-2 (-29, 6)	1 (-7, 8)	0.413
γ-GTP (U/L)	week 0	25	25	22 (17, 36)	20 (14, 29)		-	-		-	-	-
	week 12	25	22	24 (16, 32)	20.5 (15, 29)		0.390	0.553		0 (-1, 3)	0 (-1, 2)	0.898
	week 24	25	19	24 (17, 34)	21 (15, 26)		0.419	0.123		-1 (-4, 3)	1 (-1, 3)	0.161
Cr (mg/dL)	week 0	25	25	0.74 (0.66, 0.95)	0.73 (0.61, 0.91)		-	-		-	-	-
	week 12	25	22	0.80 (0.65, 0.98)	0.79 (0.67, 0.933)		0.089	0.211		0.03 (-0.04, 0.09)	0.03 (-0.04, 0.08)	0.873
	week 24	25	19	0.79 (0.63, 0.92)	0.70 (0.64, 1.01)		0.742	0.601		-0.02 (-0.05, 0.03)	0.01 (-0.04, 0.07)	0.476
e-GFR (mL/min/1.73 m²)	week 0	25	25	63.6 (58.2, 77.5)	64.9 (56.8, 81.5)		-	-		-	-	-
	week 12	25	22	64.1 (55.3, 71.0)	62.7 (49.2, 77.3)		0.259	0.032*		-2.3 (-7.8, 2.8)	-2.8 (-8.8, 1.5)	0.488
	week 24	25	19	66 (59.7, 81.1)	63.2 (52.6, 73.6)		0.872	0.647		1.4 (-4.0, 4.0)	-1.5 (-4.7, 4.1)	0.636
Na (mmol/L)	week 0	25	25	141.2 (140.4, 143.1)	141.3 (139.8, 142.8)		-	-		-	-	-
	week 12	25	22	141.8 (140.1, 142.5)	140.9 (138.5, 142.2)		0.737	0.559		0.3 (-1.5, 1.3)	-0.3 (-2.4, 1.7)	0.815

	week 24	25	19	142.0 (140.4, 142.9)	141.2 (138.6, 142.7)		0.554	0.324		0.4 (-1.4, 1.8)	-0.6 (-3.2, 1.8)	0.270
K (mmol/L)	week 0	25	25	4.3 (4.0, 4.4)	4.2 (4.1, 4.4)		-	-		-	-	-
	week 12	25	22	4.3 (4.0, 4.6)	4.5 (4.2, 4.7)		0.820	0.134		0.0 (-0.2, 0.1)	0.3 (-0.1, 0.3)	0.209
	week 24	25	19	4.2 (3.8, 4.4)	4.4 (4.2, 4.5)		0.354	0.244		0.0 (-0.4, 0.2)	0.1 (0.0, 0.2)	0.156
Ca (mg/dL)	week 0	25	25	9.5 (9.2, 9.8)	9.6 (9.2, 9.9)		-	-		-	-	-
	week 12	25	22	9.4 (9.1, 9.7)	9.5 (9.2, 9.7)		0.615	0.636		0.1 (-0.4, 0.1)	-0.1 (-0.3, 0.1)	0.789
	week 24	25	19	9.5 (9.0, 9.6)	9.4 (9.3, 9.5)		0.029*	0.326		-0.2 (-0.3, 0.1)	0.0 (-0.4, 0.2)	0.617
P (mg/dL)	week 0	25	25	3.7 (3.5, 3.9)	3.7 (3.2, 3.8)		-	-		-	-	-
	week 12	25	22	3.7 (3.1, 4.0)	3.6 (3.3, 3.9)		1.000	0.765		0.1 (-0.4, 0.5)	0.1 (-0.4, 0.5)	0.765
	week 24	25	19	3.4 (3.2, 3.7)	3.5 (3.2, 3.8)		0.0318*	0.586		-0.3 (-0.5, 0.1)	0.1 (-0.3, 0.4)	0.085
WBC (10^9/L)	week 0	25	25	5.5 (5.1, 6.5)	5.4 (4.9, 7.1)		-	-		-	-	-
	week 12	25	22	5.7 (4.9, 6.1)	5.9 (5.2, 7.4)		0.206	0.961		-0.2 (-0.5, 0.3)	-0.1 (-0.7, 0.6)	0.522
	week 24	25	19	5.6 (4.6, 6.2)	5.6 (5.0, 6.5)		0.237	0.809		-0.1 (-0.8, 0.3)	0.3 (-1.0, 0.7)	0.660

RBC (10^12/L)	week 0	25	25	4.13 (3.83, 4.46)	4.14 (3.78, 4.31)		-	-		-	-	-
	week 12	25	22	4.14 (3.91, 4.33)	4.07 (3.71, 4.37)		1.000	0.060		0.03 (-0.20, 0.21)	-0.09 (-0.22, 0.06)	0.232
	week 24	25	19	4.11 (3.94, 4.40)	4.12 (3.80, 4.43)		0.484	0.825		0.03 (-0.11, 0.22)	0.05 (-0.15, 0.14)	0.696
Neutrophil (%)	week 0	25	25	56.8 (53.0, 65.5)	65.0 (57.2, 70.4)		-	-		-	-	-
	week 12	25	22	57.4 (51.8, 66.7)	64.9 (61.4, 69.1)		0.757	0.548		-1.3 (-2.6, 5.5)	-0.8 (-3.7, 4.0)	0.662
	week 24	25	19	60.3 (53.2, 65.3)	66.8 (61.2, 69.7)		0.731	0.891		-0.2 (-3.4, 6.6)	0.3 (-3.7, 3.7)	0.850
lymphocytes (%)	week 0	25	25	31.9 (26.1, 35.8)	25.4 (22.6, 30.1)		-	-		-	-	-
	week 12	25	22	33.1 (24.5, 35.3)	23.4 (21.4, 30.2)		0.696	0.987		0.3 (-4.1, 2.0)	0.0 (-3.2, 4.4)	0.798
	week 24	25	19	28.8 (24.8, 34.9)	24.0 (20.3, 26.3)		0.247	0.513		-0.7 (-4.2, 2.1)	0.0 (-5.9, 1.5)	0.705
Monocyte (%)	week 0	25	25	6.7 (5.1, 7.7)	6.6 (5.8, 7.4)		-	-		-	-	-
	week 12	25	22	7.0 (5.4, 8.2)	6.7 (5.1, 7.8)		0.116	0.884		0.2 (-0.2, 1.0)	-0.1 (-0.8, 0.9)	0.220
	week 24	25	19	6.6 (4.9, 8.3)	6.8 (5.0, 8.5)		0.389	0.673		0.4 (-0.5, 1.0)	0.3 (-0.7, 0.9)	0.840

Eosinophil (%)	week 0	25	25	2.3 (1.6, 4.9)	2.1 (1.0, 2.9)		-	-		-	-	-
	week 12	25	22	2.3 (1.4, 4.0)	2.4 (1.1, 4.0)		0.299	0.053		0.0 (-0.9, 0.3)	0.6 (-0.1, 1.0)	0.014*
	week 24	25	19	2.7 (1.7, 4.7)	2.5 (1.3, 3.8)		0.886	0.212		-0.1 (-0.6, 1.0)	0.2 (-0.4, 1.0)	0.407
Basophil (%)	week 0	25	25	0.6 (0.4, 0.7)	0.3 (0.2, 0.7)		-	-		-	-	-
	week 12	25	22	0.6 (0.5, 0.7)	0.4 (0.3, 0.7)		0.661	0.326		0.1 (-0.2, 0.2)	0.1 (0.0, 0.2)	0.923
	week 24	25	19	0.6 (0.5, 0.8)	0.5 (0.4, 0.6)		0.454	0.776		0.0 (-0.2, 0.2)	0.0 (-0.2, 0.2)	0.802
LDH (U/L)	week 0	25	25	181 (165, 216)	190 (172, 212)		-	-		-	-	-
	week 12	25	22	186 (164, 211)	192 (169, 216)		0.872	0.198		-1 (-11, 15)	-5 (-16, 4)	0.418
	week 24	25	19	182 (176, 217)	178 (162, 209)		0.527	0.904		4 (-10, 21)	-3 (-16, 15)	0.627
Ch-E (U/L)	week 0	25	25	271 (221, 300)	270 (238, 286)		-	-		-	-	-
	week 12	25	22	273 (224, 288)	270 (226, 313)		0.872	0.733		-2 (-17, 31)	-6 (-21, 17)	0.558
	week 24	25	19	272 (208, 314)	279 (233, 317)		0.270	0.573		5 (-9, 27)	8 (-19, 27)	0.887
CPK (U/L)	week 0	25	25	86 (58, 115)	79 (64, 109)		-	-		-	-	-

	week 12	25	22	80 (64, 116)	90 (61, 125)		0.154	0.210		-5 (-20, 6)	-8 (-17, 6)	0.966
	week 24	25	19	90 (68, 126)	88 (58, 126)		0.732	0.601		0 (-7, 15)	-3 (-29, 19)	0.455
BUN (mg/dL)	week 0	25	25	20 (15, 23)	19 (16, 23)		-	-		-	-	-
	week 12	25	22	18 (15, 23)	20 (15, 24)		0.961	0.922		0 (-3, 2)	-1 (-3, 3)	0.864
	week 24	25	19	18 (14, 22)	20 (16, 24)		0.625	0.602		-1 (-3, 2)	0 (-5, 3)	0.712
TP (g/dL)	week 0	25	25	6.8 (6.5, 7.2)	6.9 (6.7, 7.1)		-	-		-	-	-
	week 12	25	22	6.8 (6.6, 7.1)	6.9 (6.3, 7.2)		1.000	0.600		0.0 (-0.2, 0.3)	0.0 (-0.4, 0.2)	0.623
	week 24	25	19	6.9 (6.7, 7.3)	6.8 (6.6, 7.2)		0.313	0.849		0.0 (-0.2, 0.4)	0.0 (-0.3, 0.3)	0.378
Albumin (g/dL)	week 0	25	25	4.1 (3.8, 4.2)	4.2 (3.8, 4.3)		-	-		-	-	-
	week 12	25	22	4.0 (3.8, 4.3)	4.0 (3.8, 4.1)		0.837	0.02*		0.0 (-0.1, 0.2)	-0.2 (-0.3, 0.1)	0.065
	week 24	25	19	4.0 (3.8, 4.4)	4.1 (4.0, 4.3)		0.527	0.169		0.0 (-0.1, 0.2)	-0.1 (-0.3, 0.1)	0.181
T-cho (mg/dL)	week 0	25	25	184 (161, 204)	195 (177, 205)		-	-		-	-	-
	week 12	25	22	181 (162, 197)	188 (162, 211)		0.208	0.178		-5 (-21, 9)	-1 (-19, 9)	0.890
	week 24	25	19	178 (165, 202)	194 (171, 206)		0.619	0.695		-4 (-15, 15)	-1 (-13, 9)	0.953
HDL-C (mg/dL)	week 0	25	25	56 (51, 73)	57 (50, 76)		-	-		-	-	-
	week 12	25	22	55 (49, 68)	60 (48, 70)		0.594	0.454		-1 (-5, 5)	-1 (-7, 4)	0.949

	week 24	25	19	56 (48, 70)	59 (50, 67)		0.840	0.643		1 (-5, 4)	-1 (-6, 5)	0.877
LDL (mg/dL)	week 0	25	25	99 (87, 113)	108 (96, 121)		-	-		-	-	-
	week 12	25	22	92 (80, 110)	105 (86, 119)		0.157	0.444		-3 (-15, 8)	-4 (-13, 11)	0.677
	week 24	25	19	87 (82, 111)	103 (94, 118)		0.467	1.000		-4 (-14, 12)	1 (-12, 8)	0.515
TG (mg/dL)	week 0	25	25	124 (88, 170)	136 (81, 165)		-	-		-	-	-
	week 12	25	22	125 (83, 179)	113.5 (89, 155)		0.925	0.673		6 (-37, 32)	-2 (-25, 20)	0.565
	week 24	25	19	113 (77, 138)	143 (102, 167)		0.254	0.586		-11 (-35, 12)	8 (-14, 29)	0.297
Glucose (mg/dL)	week 0	25	25	113 (99, 133)	116 (109, 135)		-	-		-	-	-
	week 12	25	22	111 (99, 125)	110 (100, 159)		0.338	0.795		-1 (-30, 11)	-4 (-12, 22)	0.436
	week 24	25	19	120 (108, 139)	120 (104, 148)		0.511	0.984		0 (-8, 14)	-2 (-19, 16)	0.484
Hemoglobin (g/dL)	week 0	25	25	13.1 (11.9, 13.8)	12.7 (11.6, 13.5)		-	-		-	-	-
	week 12	25	22	13.0 (12.4, 13.6)	12.3 (10.8, 13.3)		0.678	0.025*		0.1 (-0.4, 0.6)	-0.3 (-0.8, -0.1)	0.085

	week 24	25	19	13.2 (12.2, 13.6)	11.8 (11.2, 13.0)		0.346	0.354		0.1 (-0.3, 0.7)	-0.2 (-0.9, 0.5)	0.230
Fe (μg/dL)	week 0	25	25	85 (55, 106)	60 (46, 83)		-	-		-	-	-
	week 12	25	22	81 (66, 102)	74 (46, 85)		0.830	0.858		0 (-13, 14)	-2 (-8, 6)	0.773
	week 24	25	19	77 (55, 112)	67 (50, 74)		0.360	1.000		-11 (-23, 17)	1 (-12, 12)	0.394
TIBC (μg/dL)	week 0	25	25	321 (272, 361)	296 (265, 374)		-	-		-	-	-
	week 12	25	22	299 (275, 349)	312 (289, 380)		0.752	0.385		5 (-16, 11)	2 (-5, 20)	0.949
	week 24	25	19	308 (288, 345)	313 (284, 387)		0.577	1.000		0 (-7, 19)	3 (-23, 17)	0.803
ferritin (ng/mL)	week 0	25	25	72.5 (32.4, 153.9)	79.9 (29.7, 142.0)		-	-		-	-	-
	week 12	25	22	97.2 (64.3, 155.5)	81.5 (42.2, 127.4)		0.474	0.248		0.1 (-15.8, 33.6)	-4.7 (-11.1, 3.1)	0.316
	week 24	25	19	94.9 (30.2, 169.3)	66.2 (46.4, 139.3)		0.508	0.568		-3.8 (-22.2, 18.2)	-5.8 (-16.6, 16.7)	0.906
CPR (C-peptide immunoreactivity) (ng/mL)	week 0	25	25	3.4 (2.3, 5.6)	4.0 (2.8, 5.7)		-	-		-	-	-
	week 12	25	22	3.0 (2.5, 5.5)	4.0 (2.7, 6.8)		0.791	0.656		0.0 (-1.1, 0.9)	0.0 (-0.9, 1.6)	0.515
	week 24	25	19	4.1 (2.9, 5.6)	3.6 (2.7, 6.3)		0.751	0.953		0.1 (-1.1, 1.1)	-0.3 (-1.4, 1.1)	0.972
TSH (mIU/mL)	week 0	25	25	1.6 (1.1, 2.6)	1.6 (1.1, 2.3)		-	-		-	-	-

	week 12	25	22	1.5 (1.1, 1.8)	1.6 (1.2, 2.3)		0.101	0.862		-0.1 (-1.1, 0.2)	-0.0 (-0.2, 0.3)	0.267
	week 24	25	19	1.4 (1.2, 2.0)	2.1 (1.0, 2.6)		0.451	0.601		-0.0 (-0.7, 0.3)	0.1 (-0.3, 0.5)	0.362
fT3 (pg/mL)	week 0	25	25	2.6 (2.5, 2.8)	2.6 (2.4, 2.8)		-	-		-	-	-
	week 12	25	22	2.7 (2.4, 2.9)	2.7 (2.5, 2.9)		0.250	0.257		0.1 (-0.1, 0.3)	0.1 (-0.1, 0.2)	0.915
	week 24	25	19	2.7 (2.5, 2.9)	2.6 (2.3, 2.9)		0.352	0.938		0.0 (-0.1, 0.4)	0.0 (-0.2, 0.2)	0.608
fT4 (ng/mL)	week 0	25	25	1.3 (1.2, 1.3)	1.2 (1.1, 1.4)		-	-		-	-	-
	week 12	25	22	1.3 (1.2, 1.4)	1.3 (1.2, 1.5)		0.362	0.085		0.0 (-0.1, 0.1)	0.1 (0.0, 0.1)	0.490
	week 24	25	19	1.3 (1.2, 1.4)	1.2 (1.2, 1.4)		0.529	0.693		0.0 (-0.1, 0.1)	0.0 (-0.1, 0.1)	0.669
IL-6 (pg/mL)	week 0	25	25	2.7 (1.9, 4.9)	4 (2.3, 7.4)		-	-		-	-	-
	week 12	25	22	2.6 (1.8, 4.9)	3.5 (2.4, 5.5)		0.721	0.357		0.0 (-1.0, 0.5)	-0.4 (-2.2, 1.0)	0.423
	week 24	25	19	2.2 (1.5, 3.9)	3.5 (2.4, 6.5)		0.232	0.794		-0.2 (-1.8, 0.3)	-0.5 (-0.7, 1.1)	0.553
testosterone (ng/mL)	week 0	25	25	0.2 (0.1, 2.6)	0.2 (0.1, 3.1)		-	-		-	-	-
	week 12	25	22	0.1 (0.0, 3.2)	0.2 (0.1, 2.3)		0.420	0.687		0.0 (-0.1, 0.0)	0.0 (-0.02, 0.05)	0.458
	week 24	25	19	0.2 (0.0, 2.7)	0.2 (0.1, 2.2)		0.737	0.887		0.0 (-0.1,	0.0 (-0.05,	0.943

										0.1)	0.02)	
C-reactive protein (CRP) (mg/dL)	week 0	25	25	0.08 (0.05, 0.16)	0.11 (0.07, 0.23)		-	-		-	-	-
	week 12	25	22	0.07 (0.04, 0.22)	0.15 (0.08, 0.27)		0.513	0.422		-0.01 (-0.04, 0.02)	0.00 (-0.06, 0.09)	0.468
	week 24	25	19	0.06 (0.04, 0.18)	0.10 (0.05, 0.23)		0.563	0.267		-0.01 (-0.06, 0.00)	-0.02 (-0.10, 0.01)	0.609
TRACP-5b (mU/dL)	week 0	25	25	292 (222, 456)	255 (125, 429)		-	-		-	-	-
	week 12	25	22	344 (270, 431)	262 (145, 473)		0.853	0.506		1 (-63, 77)	21 (-40, 46)	0.709
	week 24	25	19	361 (261, 515)	272 (173.5, 443)		0.893	0.182		1 (-71, 72)	17 (-37, 112)	0.434
Total PINP (ng/mL)	week 0	25	25	39.9 (31.1, 54.1)	33.7 (22.3, 49.6)		-	-		-	-	-
	week 12	25	22	34.9 (29.1, 52.0)	33.8 (23.2, 49.2)		0.211	0.153		-1.8 (-9.3, 2.6)	1.0 (-4.2, 8.3)	0.084
	week 24	25	19	44.4 (29.0, 62.1)	31.4 (18.3, 44.8)		0.771	0.560		1.2 (-15.5, 12.0)	0.6 (-5.5, 9.8)	0.776

Regional DXA non-fat mass, thyroid hormones, testosterone, CRP, and IL-6 relocated from the original Table 3, followed by bone, oral/GI, hematological, and biochemical indices. Data are presented as median (interquartile range). N indicates the number of participants with evaluable data at each time point and may vary because of missing observations, discontinuation, or loss to follow-up. Statistical significance is indicated as follows: * nominal $p < 0.05$; ** nominal $p < 0.01$, Wilcoxon rank-sum test between groups and Wilcoxon signed-rank test within groups. No adjustment for multiple comparisons was

performed; all p-values are nominal and exploratory and should not be interpreted as confirmatory evidence.
