# Supplementary material for: Effects of Heat-Killed Bifidobacterium breve B-3, a Postbiotic, on Muscle Mass-Related Parameters in Patients with Sarcopenia: A Randomized, Double-Blind, Placebo-Controlled Pilot Trial
Source: Nutrients. 2026 Sep 20;18(18):3074. doi: 10.3390/nu18183074 (PMC13610449; doi:10.3390/nu18183074)
Supplement: Supplementary file 1 [file nutrients-18-03074-s001.zip › Table S2.pdf]

Table S2. Alpha diversity of fecal mi-crobiota in the placebo and B-3HK groups.

		week 0				week 24		
		Placebo (N=24)	B-3HK (N=23)	P-value		Placebo (N=23)	B-3HK (N=15)	P-value
**Fecal microbiota**								
	Shannon	5.7 (5.33, 6.13)	5.6 (5.23, 6.08)	0.710		5.7 (5.16, 6.08)	5.7 (5.51, 5.87)	0.905
	Chao1	209.0 (150.75, 251.00)	196.0 (155.50, 247.05)	0.907		195.0 (147.50, 217.00)	190.0 (165.00, 241.52)	0.654
	Evenness	0.76 (0.729, 0.782)	0.75 (0.733, 0.774)	0.647		0.75 (0.704, 0.780)	0.74 (0.724, 0.761)	0.788
	Faith_pd	25.0 (21.25, 27.60)	22.8 (21.44, 27.56)	0.840		23.9 (20.65, 27.39)	21.8 (21.21, 26.29)	0.633
	Observed OTUs	205.0 (147.25, 242.50)	188.0 (150.00, 243.50)	0.958		193.0 (144.00, 211.00)	190.0 (163.00, 223.50)	0.654

Data are presented as median (interquartile range). N indicates the number of participants with evaluable data at each time point and may vary because of missing observations, discontinuation, or loss to follow-up. The Wilcoxon rank-sum test was used to analyze differences between groups. No adjustment for multiple comparisons was performed; all p-values are nominal and exploratory and should not be interpreted as confirmatory evidence.
