# Supplementary material for: Effects of Heat-Killed Bifidobacterium breve B-3, a Postbiotic, on Muscle Mass-Related Parameters in Patients with Sarcopenia: A Randomized, Double-Blind, Placebo-Controlled Pilot Trial
Source: Nutrients. 2026 Sep 20;18(18):3074. doi: 10.3390/nu18183074 (PMC13610449; doi:10.3390/nu18183074)
Supplement: Supplementary file 1 [file nutrients-18-03074-s001.zip › Table S3.pdf]

Table S3. Composition of the fecal microbiota at the phylum and genus levels in the placebo and B-3HK groups.

		week 0				week 24		
		Placebo (N=24)	B-3HK (N=23)	P-value		Placebo (N=24)	B-3HK (N=23)	P-value
**Phylum**								
	Actinobacteriota	4.1 (1.0, 9.6)	3.6 (2.3, 5.7)	0.73		3.3 (1.1, 9.4)	4.3 (1.6, 9.0)	0.72
	Bacteroidota	28.8 (20.2, 34.4)	29.4 (22.3, 37.1)	0.77		23.6 (17.3, 32.1)	26.7 (22.6, 36.4)	0.40
	Firmicutes_A	47.2 (36.3, 54.0)	37.3 (34.1, 49.4)	0.46		43.3 (31.9, 53.5)	37.1 (30.2, 48.9)	0.51
	Firmicutes_D	7.0 (3.1, 11.0)	5.3 (2.8, 11.6)	0.79		6.6 (3.8, 13.7)	6.6 (4.3, 9.3)	0.95
	Proteobacteria	3.4 (1.3, 10.0)	3.6 (1.0, 8.8)	0.74		4.4 (1.3, 7.9)	4.6 (1.0, 12.4)	0.86
	unknown	1.2 (0.5, 6.6)	1.0 (0.3, 3.0)	0.29		3.0 (0.7, 9.4)	1.8 (0.4, 3.4)	0.18
**Genus**								
	Bifidobacterium_388775	3.7 (0.8, 7.3)	3.2 (1.4, 5.3)	0.59		3.0 (1.0, 9.2)	3.9 (1.3, 8.8)	0.83
	Bacteroides_H	9.6 (5.4, 12.1)	10.6 (6.3, 15.7)	0.69		7.8 (5.3, 12.6)	10.3 (6.0, 15.8)	0.68
	Phocaeicola_A_858004	7.4 (2.7, 13.6)	7.0 (5.2, 13.6)	0.87		5.9 (3.0, 9.7)	10.4 (4.1, 20.2)	0.16
	Parabacteroides_B_862066	1.8 (1.2, 3.5)	2.1 (1.1, 4.5)	0.89		2.1 (0.9, 3.6)	1.4 (0.8, 2.9)	0.39
	unclassified Lachnospiraceae	0.5 (0.2, 1.2)	1.0 (0.4, 2.2)	0.18		0.3 (0.2, 1.7)	0.7 (0.5, 1.2)	0.27
	Agathobacter_164117	0.3 (0.2, 1.4)	0.5 (0.1, 2.9)	0.86		0.7 (0.2, 2.0)	1.7 (0.3, 4.6)	0.24
	Blautia_A_141781	4.3 (2.4, 5.3)	3.5 (2.4, 5.2)	0.66		4.0 (2.8, 5.2)	3.4 (1.9, 6.0)	0.59
	Lachnospira	0.9 (0.1, 1.6)	0.5 (0.2, 1.5)	0.91		0.7 (0.0, 1.9)	1.3 (0.6, 1.9)	0.70
	Ruminococcus_E	0.7 (0.1, 4.2)	1.4 (0.2, 3.6)	0.91		0.8 (0.1, 4.6)	0.2 (0.1, 3.2)	0.88
	Faecalibacterium	6.6 (0.7, 11.5)	5.3 (0.7, 9.5)	0.66		6.4 (1.1, 13.2)	4.6 (2.0, 8.4)	0.93

	Gemmiger_A_73129	1.3 (0.2, 3.7)	0.2 (0.1, 2.5)	0.16		0.9 (0.1, 2.9)	0.2 (0.1, 2.8)	0.71
	Streptococcus	2.6 (0.7, 5.5)	1.5 (0.9, 4.8)	0.77		3.1 (0.5, 6.5)	1.7 (0.9, 5.2)	0.83
	unknown	1.2 (0.5, 6.6)	1.0 (0.3, 3.0)	0.29		3.0 (0.7, 9.4)	1.8 (0.4, 3.4)	0.18

Data are presented as the medians (interquartile ranges) for taxa with a median relative abundance greater than 1% in at least one group. N indicates the number of participants with evaluable data at each time point and may vary because of missing observations, discontinuation, or loss to follow-up. The Wilcoxon rank-sum test was used to analyze differences between groups. * $p < 0.05$. No adjustment for multiple comparisons was performed; all p-values are nominal and exploratory and should not be interpreted as confirmatory evidence.
